# Whole-genome sequencing cluster analysis reveals complex healthcare-associated COVID-19 dynamics

**DOI:** 10.1017/ash.2023.341

**Published:** 2023-09-29

**Authors:** Theodore Rader, Graham Snyder, Lee Harrison, Vatsala Rangachar Srinivasa, Marissa Griffith, Lora Pless, Ashley Chung

## Abstract

**Background:** Identifying and interrupting transmission of severe acute respiratory syndrome coronavirus 2 and resulting disease (COVID-19) in acute-care settings can be challenging due to incubation period, asymptomatic infection, and prevalent community disease. To elucidate routes of infection and interrupt COVID-19 outbreaks with uncertain epidemiological chains of transmission, UPMC utilized reactive whole-genome sequencing (WGS) of viral specimens. **Methods:** UPMC infection prevention teams identified healthcare-associated COVID-19 clusters with uncertain transmission pathways among patients and/or healthcare personnel (HCP) in acute-care hospitals. Nasopharyngeal samples preserved in viral transport media were obtained for genetic analyses. Nucleic acids were extracted and WGS libraries were prepared by targeted enrichment or multiplex PCR methodologies. Resulting sequencing reads were aligned to the Wuhan-1 reference genome, followed by identification of single-nucleotide polymorphisms (SNPs) among the genomes and construction of a phylogenetic tree. Specimens were considered genetically similar if there were ≤2 SNP differences between viral genomes within a cluster. **Results:** Between May 2020 until August 2022, infection prevention teams requested WGS for 17 healthcare-associated clusters of COVID-19 involving 182 individuals across 8 UPMC facilities (median outbreak size, 9 individuals; range, 2–26). Of the 182 individuals, 36 lacked clinical specimens and 30 did not pass WGS quality-control criteria of ≥95% of the reference genome with a minimum of 10× coverage. Of the 116 sequenced genomes, 94 (81%) had virus genetically similar to ≥1 other specimen, including 87 (83.6%) of 104 patient viruses and 7 (58.3%) of 12 HCP viruses, comprising 22 clusters (Fig. 1). The remaining 22 (20.6%) specimens were genetically unrelated. In total, 16 (94.1%) of the 17 epidemiologically identified clusters had 2 or more individuals with a genetically similar virus. Also, 7 (41.1%) of these clusters had genetically similar viral genomes for every individual within each cluster. Also, 9 (52.9%) clusters contained both genetically related and unrelated specimens: 5 of these had more complex genomic profiles (including 4 clusters containing 2 distinct subclusters of ≥2 genetically related viruses) and 1 cluster contained 3 subclusters of ≥2 genetically related viruses. In the outbreak with 3 clusters, 3 SNPs separated specimens from 2 temporally proximal clusters, suggesting possible propagation between clusters (cluster B-3 in Fig. 1). **Conclusions:** WGS can complement traditional epidemiological investigations of healthcare-associated COVID-19 outbreaks, revealing complex transmission dynamics. Future investigations will characterize the impact of WGS on determining specific transmission pathways in acute-care facilities.

**Figure:**
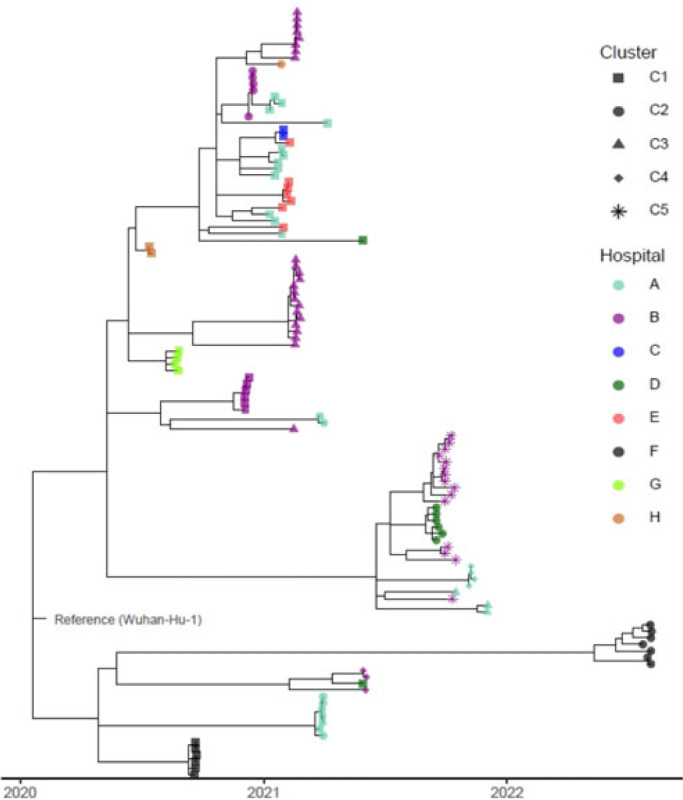

**Disclosures:** None

